# How temperature tunes muscle mechanics during eccentric contractions

**DOI:** 10.1152/ajpcell.00047.2026

**Published:** 2026-03-09

**Authors:** Graham N. Askew, Roger W.P. Kissane

**Affiliations:** 1 School of Biomedical Sciences, University of Leeds, UK; 2 Department of Musculoskeletal & Ageing Science, University of Liverpool, The William Henry Duncan Building, 6 West Derby Street, Liverpool L7 8TX, UK

**Keywords:** Eccentric Contraction, Force Velocity, Hill-Type Model, Muscle Mechanics, Titin

## Abstract

Eccentric muscle contractions occur when muscles actively lengthen, acting as brakes that dissipate energy and stabilise joints. When actively stretched, muscle force rises in two phases: an initial steep increase (phase 1), followed by a slower, sustained rise (phase 2). The temperature sensitivity of this response is poorly understood, despite its relevance for musculoskeletal models that often rely on data collected at non-physiological temperatures. We studied active lengthening contractions in mouse extensor digitorum longus muscle at 17°C, 27°C and 37°C. Force development in both phases was temperature sensitive. Phase 1 stiffness decreased at higher temperatures, consistent with faster ATP-dependent cross-bridge detachment, and contributions from mechanical strain-dependent detachment. In phase 2, stiffness increased with temperature, consistent with stronger and faster titin-actin interactions. The transition between phases (muscle ‘*give*’) varied with temperature and may reflect lower temperatures delaying cross-bridge detachment and engagement of the parallel elastic elements. Together, these findings highlight the intrinsic tuning of muscle mechanics, with potential implications for susceptibility to muscle damage under different thermal conditions, and provide a foundation for the development for more accurate musculoskeletal models.

## Introduction

Eccentric muscle contraction is a common feature of many locomotor behaviours, such as running downhill, landing, or decelerating a load, where muscles act as brakes dissipating mechanical energy as heat (1, 2). For over 80 years it has been established that actively stretched (eccentric) skeletal muscle contractions generate a higher force than during passive stretching, or during isometric (fixed-length) or concentric (shortening) contractions (3). An actively stretched muscle presents with a dynamic force profile (4, 5) with an initial rapid and steep phase-1 response followed by a longer and shallower phase-2 response.

The rate of enzyme-catalysed biological processes is influenced by temperature (6). In contemporary cross-bridge models (7), detachment and attachment rates increase with temperature, leading to faster cross-bridge cycling and a more rapid power stroke, with implications for muscle mechanics and performance. This is evident in the extensive research demonstrating the temperature sensitivity of the concentric force-velocity relationship, where muscle shortening velocity increases with temperature across physiological ranges (8–11). In contrast, the effects of temperature on the eccentric force-velocity relationship have been much less extensively studied (12, 13). Based on current understanding of the mechanisms underlying the response to active stretching, it is expected that both the force response and eccentric force-velocity relationship will vary with temperature. The phase-1 response is believed to arise from increased strain on already attached cross-bridges (14, 15) that leads to a steep rise in force. The detachment of myosin heads during phase-1, leads to a transition into a shallower phase-2 force response (5). Phase-2 is thought to be driven by increased strain on the PEVK region of titin, which acts as an activation-dependent viscoelastic spring in parallel to the thin and thick filaments. This behaviour results from the Ca^2+^-dependent binding between titin (N2A and PEVK elements) and actin (16–18), resulting in a high, sustained force during stretch (19–22).

Computational biomechanical models are often necessary to understand complex biological systems (e.g. masticatory system (23); locomotion: (24)), to explore optimal performance (25) or to predict *in vivo* function in biomechanics (26, 27) and motor control research (28, 29). These models must incorporate a representation of the eccentric force-velocity relationship. However, the experimental data underpinning such models are frequently derived from different species and recorded at non-physiological temperatures (Appendix Fig. A1), which may limit their physiological relevance. A detailed examination of how temperature affects the eccentric force-velocity relationship is therefore essential: not only for assessing the limitations of models that use such data, but also for improving their accuracy and applicability.

Therefore, we set out to characterise the temperature dependence of the eccentric force-velocity relationship in mammalian skeletal muscle, subjecting the mouse extensor digitorum longus (EDL) muscle to eccentric ramp contractions at three temperatures: 17, 27 and 37°C. We hypothesise that: (1) the phase-1 force response will be reduced at higher temperatures due to faster cross-bridge detachment, leading to lower muscle stiffness and a slower rate of force rise; (2) the phase-2 response will be less affected by temperature, but may show increased stiffness if titin-actin binding becomes stronger or faster at higher temperatures.

## Materials and Methods

All experimental procedures complied with institutional and UK Home Office guidance. This study adheres to the ethical standards set by the journal and follows established guidelines for animal research (30).

### Animals

Twenty-four male C57B6 mice (Charles River) 8-10 weeks of age, body mass 25.7 ± 1.9g were used in this study. Animals were housed under a 12hour light:dark cycle at 21 degrees centigrade and had *ad libitum* access to food and water.

### Muscle isolation

Mice were killed using an approved schedule 1 method, as defined in the UK Animals (Scientific Procedures) Act 1986. Both hindlimbs of the mouse were removed and transferred to chilled (4°C), oxygenated (95% O_2_, 5% CO_2_) Krebs-Henseleit solution [117 NaCl, 4.7 KCl, 2.5 CaCl_2_, 1.2 MgSO_4_, 24.8 NaHCO_3_, 1.2 KH_2_PO_4_ and 11.1 glucose; concentrations in mmol L^-1^] (31). The EDL muscles were dissected free and aluminium foil clips were attached to the tendons at the proximal and distal ends of the muscles (32), so that external series tendon compliance was minimised.. Muscles were suspended vertically in a flow-through Perspex chamber filled with circulating, oxygenated Krebs–Henseleit solution. The muscles were attached to an ergometer (series 300B-LR; Aurora Scientific Inc.) via a light stainless-steel rod and muscle length was altered using a digital height gauge (Mitutoyo Corporation, Kanagawa, Japan). Muscles were left for 30 mins to thermo-equilibrate and recover from the dissection. Platinum electrodes were positioned inside the chamber parallel to the long-axis of the muscle was activated using an isolated stimulator (Universal Isolated Stimulator Output, Hugo Sachs Elektronik, Harvard Apparatus GmbH, March-Hugstetten, Germany).

### Temperature control

The EDL muscles were maintained at either 17, 27 or 37°C throughout the course of the experiment using a continuous flow of oxygenated Krebs-Henseleit solution, maintained by a refrigerated circulating water bath (LT ecocool 100, Grant Instruments (Cambridge) Ltd, Royston, Bedfordshire, UK).

### Isometric characteristics

All muscles were subjected to a series of isometric twitches (supramaximal stimulus of 0.2ms pulse) at incrementally adjusted muscle lengths to find the optimal length for maximising twitch-force generation (*L*_0_). Maximal isometric tetanic force (*P*_0_) at *L*_0_ was determined using a train of stimuli delivered at the frequency of stimulation that yielded a fully fused tetanus at each temperature. Data were sampled at 2 kHz during isometric contractions.

### Force-velocity characteristics

The concentric force-velocity relationship of the EDL was determined using a series of isotonic afterloaded contractions across a range of forces (~5-80% of P_0_) (33), with data sampled at 2 kHz. The force-velocity relationship was determined by fitting a hyperbolic-linear function (11, 22) to the data, comprising three coefficients, *A, B* and *C*. The maximal shortening velocity at zero force (*V*_max_), expressed relative to fibre length (*FL*) was calculated. Peak instantaneous isotonic power $\left({\dot{W}}_{\max}\right)$ and the power ratio $\left({\dot{W}}_{\max}\right)$, were also determined from the fitted force-velocity relationship (11).

**Figure 1:**
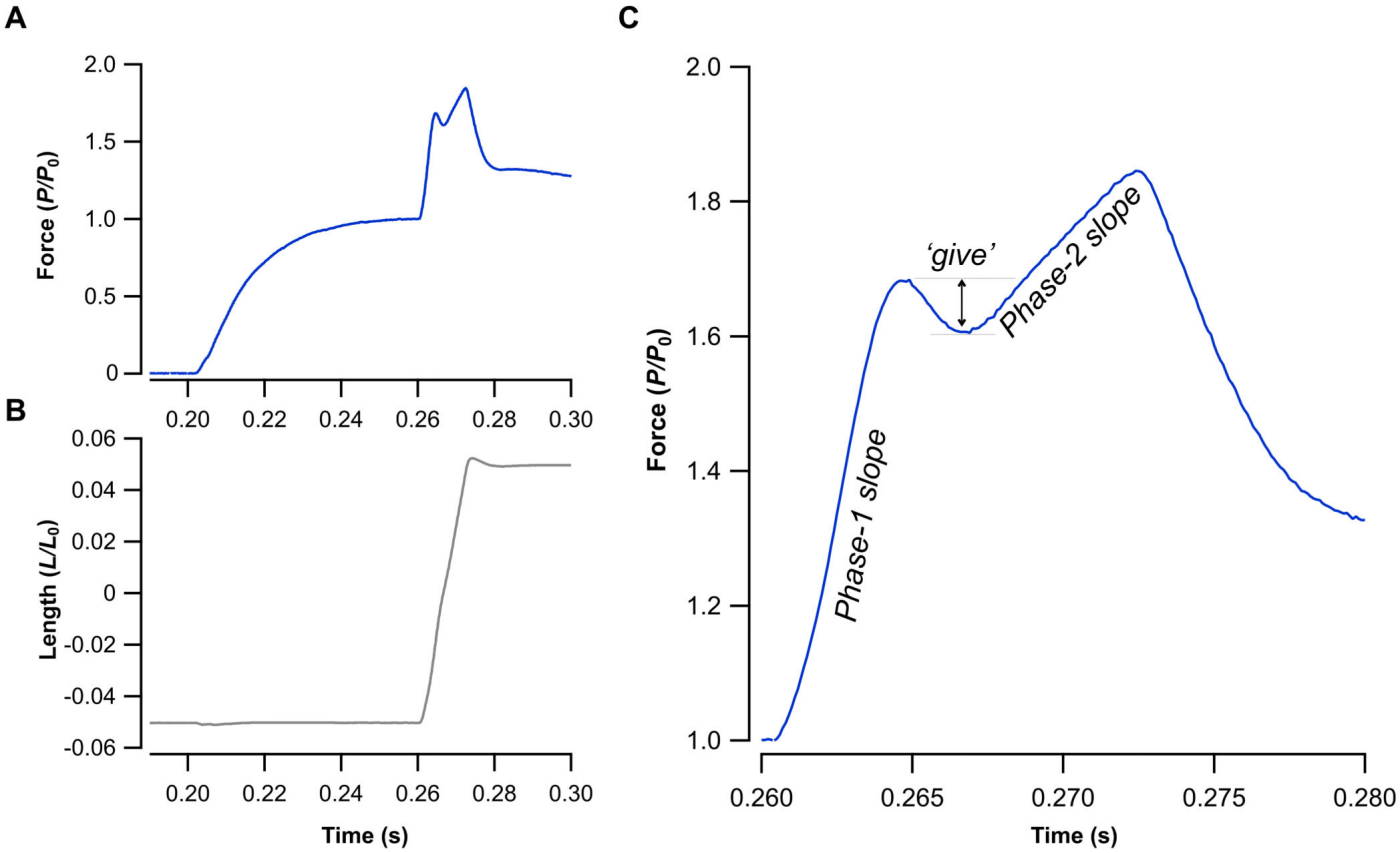
Eccentric contraction of the mouse extensor digitorum longus. Example force profile (A) of the extensor digitorum longus muscle when stretched (B) during a tetanic contraction. Magnified force trace from (A) with annotated dynamic components contained within the eccentric response (C). Phase-1 slope corresponds to the rapid rise in force development seen immediately after the initiation of stretch. After which the muscle transitions into a shallower, phase-2 response. However, at high enough velocity (and lower temperatures) muscles may present with an initial decrease in force response, prior to the steady-state phase-2 response, typically referred to as muscles ‘*give*’.

The eccentric force-velocity relationship was determined using isovelocity lengthening ramps (Fig. 1) (22). The muscle was lengthened by 1mm (~13.8-14.8% fibre strain), symmetrically spanning *L*_0_ (22). Muscles were lengthened at velocities up ~60% of temperature specific *V*_max_. Data were sampled at 5 kHz. Force and velocity were averaged across *L*_0_ (selecting 0.05mm) and used to plot the eccentric force velocity relationship. The lengthening force-velocity relationship was fit with a hyperbolic function (12, 22). Coefficients D and E were derived, representing the plateau height (D) and the curvature (E) of the relationship (22). The rate of force development (*d*P/P_0_/*dt*) was calculated across the rapid phase-1 and slower phase-2 slopes (Fig. 1). Data were excluded (and the experiment terminated) if the P_0_ in periodic control isometric tetanic contractions fell below 70% of the initial value (33–35).

Muscle ‘*give*’ was quantified using similar methods to those previously reported (36) (Fig. 1). Eccentric ramps were binarily categorised into groups that presented with or without muscle ‘*give*’, determined by the presence of a transient drop in force, below that of the transition point. In a sub-sample of the fastest relative lengthening velocities across the three temperatures (-0.45 to -0.58 *V/V*_max_), we measured the relative force (*P/P*_0_) at the transition between the linear phase-1 slope and the onset of the drop in relative force [Fig. 1; analogous to the critical point in Minozzo and Rassier (37)]. The drop in relative force during muscle give was quantified as the difference between the relative force at this transition point and the lowest point in the trough (Δ*P/P*_0_) (Fig. 1) (36).

### Statistics

All data processing was carried out and figures were plotted using Igor Pro 8 (V8.0.4.2). One-way ANOVAs were used to assess statistical significance in isotonic- and isovelocity-derived force-velocity metrics and coefficients. Where significant main effects were detected, post hoc comparisons were conducted using the Bonferroni correction and the threshold for statistical significance set to P<0.05 (completed using SPSS 28, v28.0.1.1). Linear data comparisons were completed in R (version 4.5.0) (38). Repeated measures correlations (39) were undertaken using rmcorr(). To test whether the relationship between velocity and relative force differed with temperature (17°C, 27°C, and 37°C), a linear regression model was fitted with relative force (dependent variable), velocity (continuous predictor) and temperature (categorical predictor). An interaction term between velocity and temperature was included to evaluate whether the slope of relative force with respect to velocity varied with temperature. Estimated marginal trends (slopes) of velocity within each temperature group were extracted using the emtrends() function from the emmeans() package (40). Pairwise comparisons of these slopes were performed using pairs(), with Tukey-adjusted p-values to control for multiple comparisons. All data are presented as either mean ± standard deviation or upper and lower 95% confidence intervals. Surface plots were created in Matlab (R2021a) using surf function.

## Results

### The effects of temperature on biphasic force-response active lengthening

**Figure 2:**
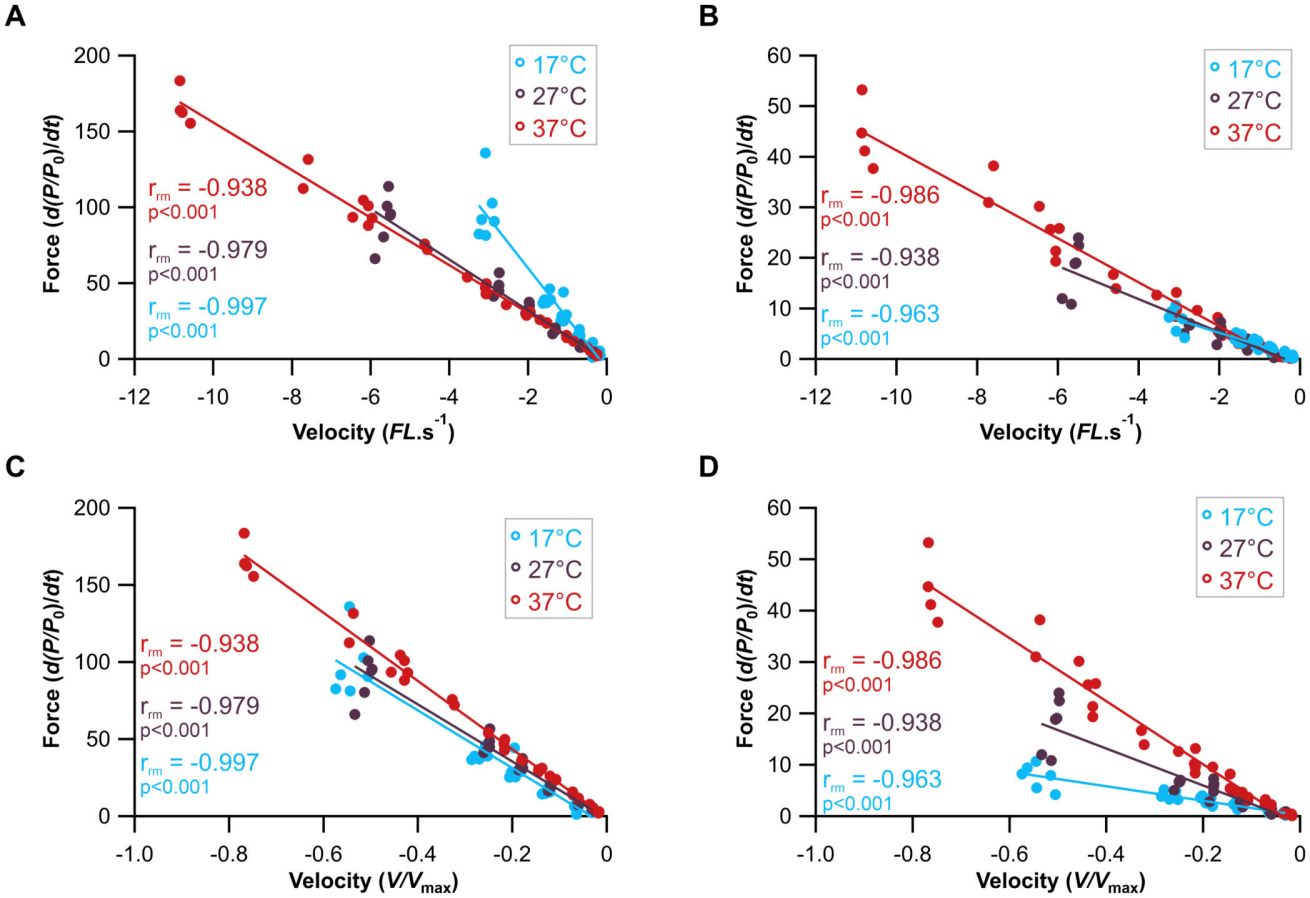
Sensitivity of the eccentric Phase-1 and Phase-2 response to temperature. The relationship between lengthening velocity and the rate of force development for Phase-1 (A, C) and Phase-2 (B, D). The relationship between absolute lengthening velocity (A-B) and velocity normalised to temperature specific V_max_ (C-D). Repeated measures correlations for 17°C (n=6), 27°C (n=5) and 37°C (n=6).

At all three temperatures there was a significant relationship between the rate of force development during phase-1 and both absolute lengthening velocity (*FL* s^-1^; Fig. 2A, Table 1) and velocity normalised to maximum shortening velocity (*V/V*_max_; Fig. 2C, Table 1): 17°C (r_rm_ (24) = -0.990, 95% CI [-0.9956, -0.9777], p<0.001, Fig. 2A, C), 27°C (r_rm_ (28) = -0.979, 95% CI [-0.9900, -0.9556], p<0.001, Fig. 2A, C) and 37°C (r_rm_ (28) = -0.997, 95% CI [-0.9988, -0.9945], p<0.001, Fig. 2A, C). While each temperature maintained a significant linear relationship between the rate of relative force development during phase-1 and lengthening velocity, the slope of this relationship differed across the temperatures (Fig. 2A, C). At 17°C, the rate of relative force development was significantly higher than at 27°C (Table 1, P<0.001) and 37°C (Table 1, P<0.001) when compared at the same absolute lengthening velocity (Fig. 2A). However, when velocity was normalised to temperature-specific *V*_max_, this relationship was reversed (Fig. 2C): the rate of relative force development at 17°C was significantly lower than at 37°C (P<0.001) and the slope of the relationship was steeper at higher temperatures (Table 1).

Similar to phase-1, at all three temperatures there was a significant relationship between the rate of relative force development during phase-2 and both absolute (Fig. 2B, Table1) and normalised (Fig. 2D, Table 1) lengthening velocity: 17°C (r_rm_ (24) = -0.963, 95% CI [-0.9835, -0.9183], p<0.001, Fig. 2B, D), 27°C (r_rm_ (28) = -0.938, 95% CI [-0.9704, -0.98726], p<0.001, Fig. 2B, D) and 37°C (r_rm_ (28) = -0.986, 95% CI [-0.9936, -0.9713], p<0.001, Fig. 2B, D). The slope of the relationship between the rate of relative force development during phase-2 and absolute lengthening velocity differed significantly with temperature (Table 1). At 17°C, the slope was significantly shallower that at 37°C, both when velocity was expressed as an absolute value (P<0.001) and when normalised to temperature specific *V*_max_ (P<0.001).

### The effects of temperature on the concentric and eccentric force-velocity relationship

**Figure 3:**
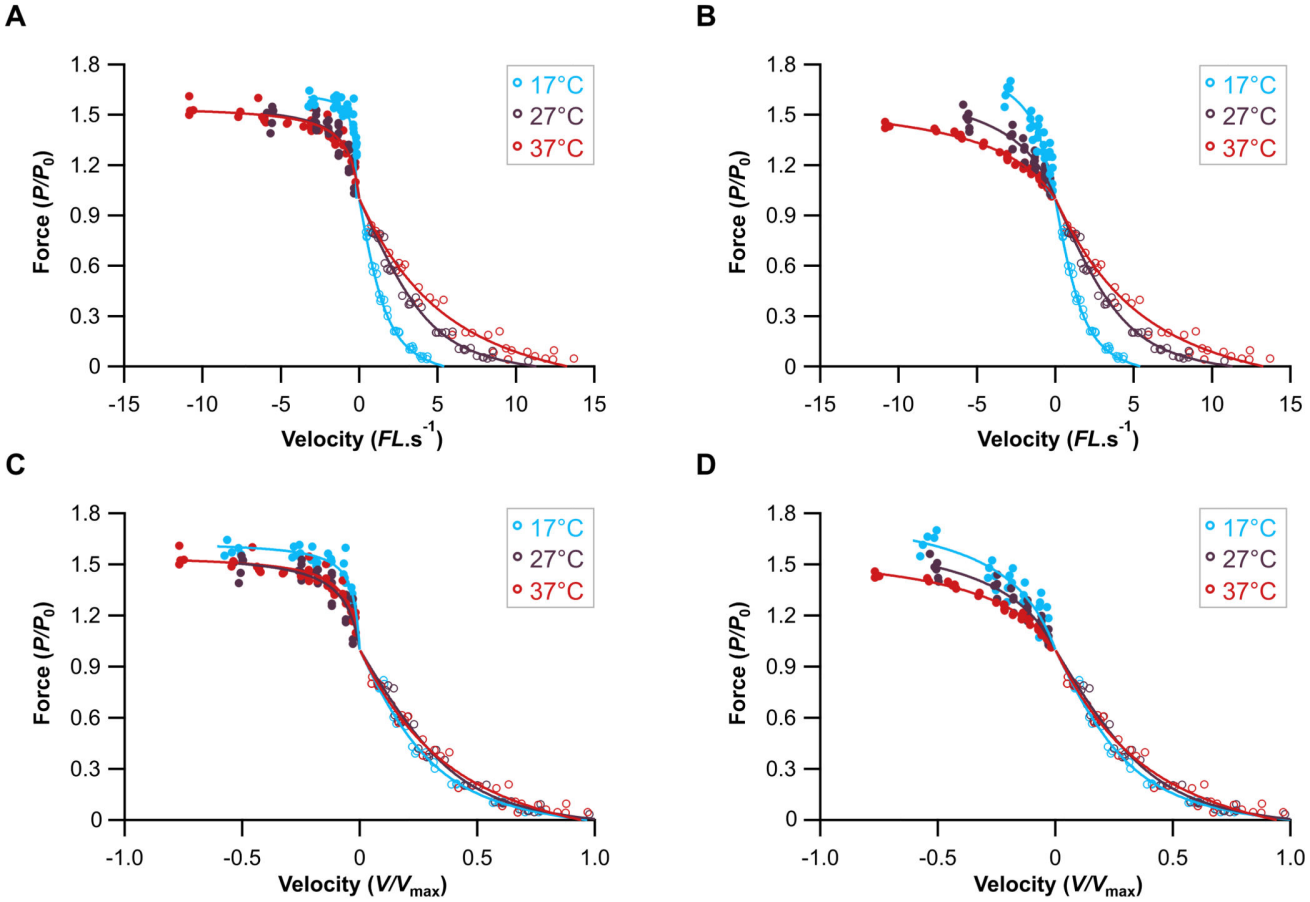
Sensitivity of the force-velocity relationship to temperature. The force-velocity relationship at 17, 27 and 37 degrees Celsius for the extensor digitorum longus muscle when measured as the muscle lengthens across L_0_ (A, C) and at the transition point between Phase-1 and Phase-2 (B, D). Given reduction seen in concentric maximum shortening velocity (V_max_) (A-B), the eccentric lengthening velocities were normalised to temperature specific V_max_ (C-D). The concentric force-velocity relationship was determined by fitting a hyperbolic-linear function (11, 22) to the data, while the eccentric force-velocity relationship was fit with a hyperbolic function (12, 22) to the 17°C (n=6), 27°C (n=5) and 37°C (n=6).

There was a significant relationship between temperature and the parameters characterising the concentric force- velocity relationship (Fig. 3). Both the maximum shortening velocity (ANOVA F_2,21_=51.727, P<0.001) and the maximum isotonic power (ANOVA F_2,21_=15.698, P<0.001, Table 2) increased significantly with increasing temperature (Table 2). There was a shift in the force-velocity relationship towards higher shortening velocities with increasing temperature (Fig. 3A, B) and the relative shortening velocity at which maximum power was generated was significantly higher at higher temperatures (ANOVA F_2,21_=6.883, P=0.005, Table 2). We have previously shown that the coefficients in Equation 2 of the eccentric curve fit correspond to distinct linear and non-linear portions of the eccentric force-velocity curve (22). Neither the D coefficient (plateau height, ANOVA F_2,13_=0.814, P=0.464, Table 3), nor the E coefficient (curvature, ANOVA F_2,13_=3.553, P=0.059, Table 3) differed significantly across the three temperatures. However, when the eccentric force-velocity relationship was fit using P/P_0_ taken at the transition between phase-1 and phase-2, temperature had a significant effect on both D (ANOVA F_2,13_=11.327, P=0.001) and E (ANOVA F_2,13_=15.646, P<0.001) coefficients (Table 3).

### The effect of temperature on muscle ‘give’

**Figure 4:**
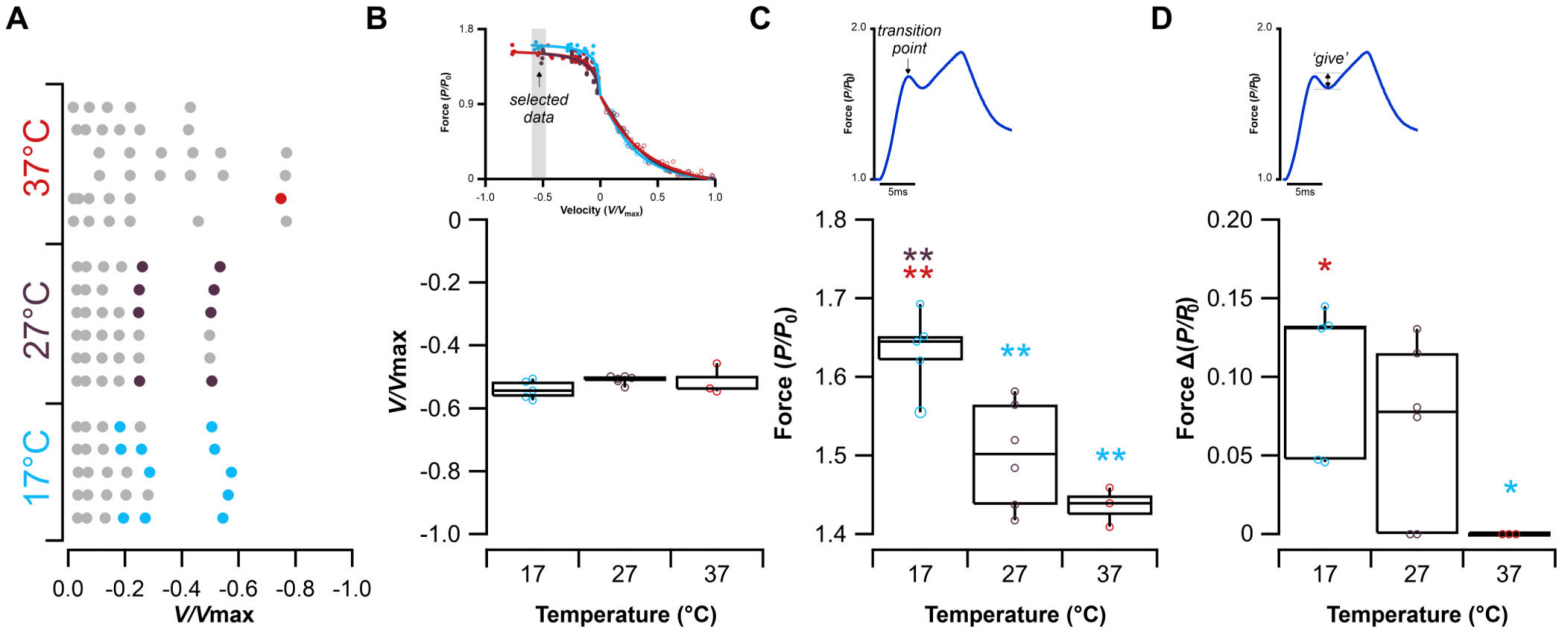
The presence of ‘give’ during eccentric ramps of the extensor digitorum longus. Scatter plot presents the *V/V*_max_ ramp applied to each muscle (rows of data) (A). Colour filled circle indicates the presence of muscle ‘*give*’, grey circles indicate no muscle ‘*give*’. The fastest stretched muscles (lengthened between -0.45 to -0.58 V/V_max_) were pooled to allow for quantitative comparisons of muscle ‘*give*’ (B). The transition point between phase-1 and phase-2 (C) and the amount of muscle ‘*give*’ (D) show significant temperature dependence. One-way ANOVAs were used to assess statistical significance, where significant main effects were detected, post hoc comparisons were conducted using the Bonferroni correction and the threshold for statistical significance set to P<0.05. * P<0.05, **P<0.01. 17°C (n=6), 27°C (n=5) and 37°C (n=6).

**Figure 5:**
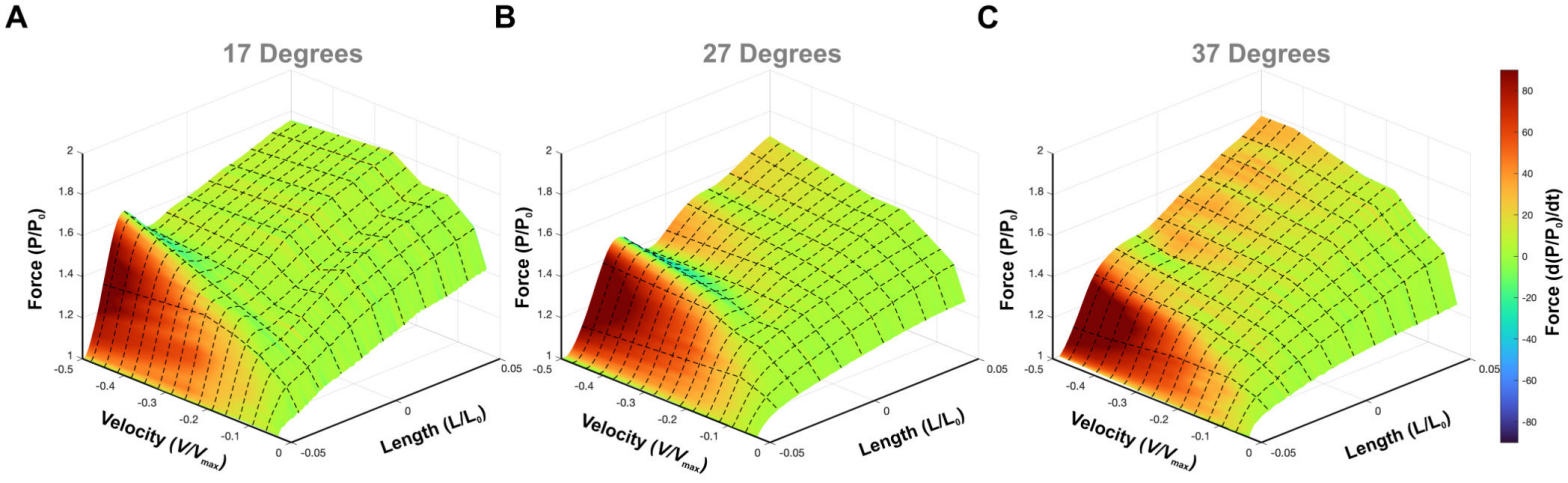
The force-length-velocity surface plot at varying temperatures on the extensor digitorum longus. The relationship between muscle lengthening velocity, and force production and length at 17°C (A), 27°C (B) and 37°C (C). Heatmap is the rate of force development.

The presence of muscle ‘*give*’ was binarily quantified for the 100 eccentric ramps performed in this experiment (Fig. 4A). Muscle ‘*give*’ occurred relatively infrequently, with the frequency decreasing as temperature increased: 17°C (11/30 ramps), 27°C (8/35 ramps) and 37°C (1/35 ramps), and appears to occur only at the higher velocities. Subsequently, a subsample of ramps in which ‘*give*’ was present, taken from the fastest range of lengthening velocities (-0.45 to -0.58 V/V_max_), was used to quantify the phenomenon. These pooled samples did not differ significantly in their lengthening velocity (ANOVA F_2,11_=1.773, P=0.215, Fig. 4B). The relative force at the transition point showed a significant temperature dependence (ANOVA F_2,11_=13.759, P=0.001, Fig. 4C), occurring at a significantly higher relative force at 17°C (1.63 ± 0.06 *P/P*_0_) compared to 27°C (1.50 ± 0.07 *P/P*_0_, P=0.007) and 37°C (1.44 ± 0.03 *P/P*_0_, P=0.001). The change in relative force between the transition point and where ‘*give*’ occurred also showed a significant temperature dependence (ANOVA F_2,11_=4.108, P=0.047, Fig. 4D). At 17°C the fall in relative force was significantly greater compared to 27°C (0.10 ± 0.05 *P/P*_0_, P=0.046) and no evidence of muscle ‘*give*’ was observed at 37°C. These data demonstrate that the occurrence of muscle ‘*give*’ is both temperature dependent and velocity dependent. A qualitative assessment of the force-length-velocity relationship of the EDL across the three temperatures (Fig. 5) indicates that the drop in force following phase-1 (i.e. muscle ‘*give*’) is greatest at the highest velocities in the 17°C (Fig. 5A) and 27°C (Fig. 5B), while across the same velocity range there is no evidence of muscle ‘*give*’ at 37°C.

## Discussion

Here we have shown, for the first time, the complex temperature dependence of the eccentric force-velocity relationship. We identify a clear dichotomy in the rate of force development during active lengthening. The rate of relative force development during phase-1 increased as temperature decreased, consistent with our hypothesis that higher temperatures accelerate ATP-dependent cross-bridge detachment, thereby reducing muscle stiffness. In contrast, the rate of relative force development during phase-2, increased with temperature, consistent with stronger and/or faster binding between titin and actin at higher temperatures, increasing the proportion of bound titin molecules. We further demonstrate that muscle ‘*give*’ not only a velocity-dependent feature of eccentric contractions but is also strongly temperature dependent. Together these findings have important implications for the interpretations of previous studies conducted at non-physiological temperatures, and for their appropriate use in the development and optimisation of musculoskeletal models.

### Thermal effects on the concentric force-velocity relationship

The overall temperature dependence observed in the concentric force-velocity relationship of our mouse EDL is similar to that reported by Rummel et al. (41) in the female, CD1 mouse EDL (Appendix Fig. A2A-B). Here, we show that with decreasing temperature, the muscle’s maximum shortening velocity decreases with Q_10_ values ranging from 1.10-1.38 closely overlapping those of Rummel, Swartz, Marsh and Faure (41) (Table 2, Appendix Fig. A2A). Isometric twitch kinetics (e.g. twitch rise time and half-relaxation time) present with the some of the greatest temperature sensitivities, with Q_10_ values as high as 4 (9, 10, 41) (Appendix Fig. A2C-D). The thermal sensitivity of the isometric twitch times and concentric force-velocity properties of mouse EDL reflect the underlying mechanisms that determine these variables. Isometric twitch kinetics are determined by temperature dependence of calcium handling, specifically the release and re-uptake of Ca^2+^ ions by the sarcoplasmic reticulum, which determines the rate of activation and relaxation, and the kinetics of the cross-bridge cycle. Similarly, the maximum shortening velocity is determined by myosin ATPase activity, which determines the rate of cross-bridge cycling, with the detachment of the myosin heads from actin being the rate-limiting step.

### Sensitivity of Phase-1 (cross-bridge dependent force production) to temperature

It is thought that the initial rapid rise in force during active muscle stretching (phase-1) results from increased strain on attached cross-bridges. We demonstrate that the relationship between the rate of relative force development and stretch velocity during phase-1 (the stiffness or the change in relative force per unit length) is temperature dependent (Fig. 2A; Table 1). The slope is steeper at cooler temperatures, indicating greater stiffness, which is evidenced by Q_10_ values below 1: 0.51 for the 17°C to 27°C range and 0.93 for the 27°C to 37°C range (Appendix Fig. A2E). This supports our first hypothesis, and suggests that at higher temperatures accelerated cross-bridge detachment, may reduce muscle stiffness. Interestingly, when the rate of relative force development is compared to stretch velocity normalised to the maximum shortening velocity of the muscle (Fig. 2C), the slope becomes shallower at lower temperatures, indicating a lower stiffness, which is evidenced by Q_10_ values above 1: 1.04 for the 17°C to 27°C range and 1.20 for the 27°C to 37°C range. If the rate of change of relative force were determined entirely by the same cross-bridge detachment kinetics that determine *V*_max_, then, under this simplifying assumption, the normalised slope (expressed relative to *V*_max_; Fig 1C) would be expected to have a Q_10_ of 1. In this conceptual framework, once temperature effects on *V*_max_ have been taken into account, any process limited by the same detachment step should become effectively temperature independent. The observation of a significant Q_10_ effect on the slope of the rate of force development in relation to normalised stretch velocity suggests that the phase-1 slope is not determined solely by ATP-driven cross-bridge detachment. Instead, it is consistent with a contribution from mechanical strain-dependent detachment processes too.

### Sensitivity of Phase-2 (non-crossbridge parallel elastic elements) to temperature

The shallower phase-2 force response during an eccentric stretch is thought to be linked to increased strain of non-crossbridge parallel elastic elements (19, 20) that has been attributed to titin behaving as an activation-dependent viscoelastic spring (21, 22, 42) whose properties depend on activation by calcium (21, 22). During phase-2, cross-bridges that detached during phase-1 rapidly reattach and contribute to the recovery of stiffness. However, the cross-bridges do not become highly strained again because the cyclical detachment and reattachment occurs at lower strains (43), so the subsequent rise in force during phase-2 occurs primarily through the viscoelastic deformation of the parallel titin spring (15).

Here we show the rate of force-development during the phase-2 component to be highly temperature dependent with significant shifts in the rate of change in force relative to lengthening velocity (Table 1; Fig. 2B, D). The Q_10_ for slope of the rate of change in force during phase-2 in relation to absolute lengthening velocity highlights the increase in muscle stiffness with increasing temperature, which is evidenced by Q_10_ values above 1: 1.43 for the 17°C to 27°C range and 1.33 for the 27°C to 37°C range (Supplement Fig. s3E). These data suggest that higher temperatures promote stronger or faster titin-actin binding (44) which contributes to increased muscle stiffness through a greater proportion of titin molecules bound to the thin filament.

### Muscle ‘give’

The phenomenon of muscle ‘*give*’ during eccentric muscle contractions has only recently begun to receive any mechanistic attention (15, 20, 36, 45, 46), despite having been identified in ramp stretches almost 50 years ago (47). Early qualitative observations in the 1990’s by Cavagna (13) and Josephson and Stokes (5) identified that the yield point (i.e. muscle ‘*give*’) occurred at high lengthening velocities in non-mammalian muscle. A recent quantitative approach by Weidner, Tomalka, Rode and Siebert (36) has shown that the onset of muscle ‘*give*’ (i.e. the transition point, *P/P*_0_, Fig. 1) is velocity dependent occurring at greater relative forces with higher lengthening velocities, which we also see in our data (Fig. 3B, D, 4). Further, Weidner, et al. (36) show that at greater lengthening velocities the force decline during muscle ‘*give*’ to be velocity dependent, again in line with findings here (Fig. 5). Our data support these previous findings that muscle ‘*give*’ is velocity dependent but further reveal that it is also temperature dependent (Fig. 4-5).

Interestingly, both Flitney and Hirst (47) and more recently Weidner, Tomalka, Rode and Siebert (36) had suggested that muscle ‘*give*’ may exhibit temperature dependence. Here, we present the first direct quantitative investigation into the temperature dependence of muscle ‘*give*’ in skeletal muscle. Specifically, we show that as temperature decreases, the relative force at which the transition between phase-1 and phase-2 occurs shifts, with the transition occurring at higher relative forces at lower temperatures (Fig. 4). In our experiments, muscle ‘*give*’ was observed at lengthening velocities as low as 20% *V*_max_, at 17°C. This aligns with previous findings in skinned fibres, where muscle ‘*give*’ was observed at velocities as low as 10% V_max_ at 11°C (20). At physiological temperatures (e.g. mammalian core temperature), muscle ‘*give*’ in the EDL was not observed until velocities exceeded 75% V_max_.

Muscle ‘*give*’ occurs during the transition between phase-1, in which resistance arises from a combination of ATP-dependent and strain-dependent cross-bridge detachment processes, and phase-2, in which activated parallel elastic elements, including titin, become engaged. This phenomenon may reflect a temporal offset between these two processes. Here we show that at lower temperatures the transition occurs at a relatively higher force, that could be due to slower cross-bridges detachment (consistent with steeper phase-1 slope of change in relative force in relation to velocity at lower temperature; Fig. 2A). The greater drop in relative force observed at lower temperatures may reflect slower engagement of the parallel elastic elements (consistent with the shallower phase-2 slope of change in relative force in relation to velocity at lower temperature; Fig. 2B).

### Functional significance of the force response to active stretch and temperature sensitivity

The functional significance of the muscle’s response to active stretch is thought to be to reduce the risk of damage and to provide a means of energy dissipation during braking activities at a low energetic cost. Phase-1 provides a rapidly attained, high magnitude resistive force due to transient stretching of attached cross-bridges. The subsequent, shallower, and sustained rise in force during phase-2 maintains force well above the isometric level, primarily through strain of the activation-dependent titin spring, which provides resistance to stretch that reduces the risk of injury and helps stabilise sarcomeres operating on the descending limb of the length-force relationship (48). At higher temperatures, muscle contraction kinetics and movement velocities are more rapid, and the potential for eccentric loading is therefore higher. The more rapid engagement of the titin-based stabilisation mechanism at higher temperatures may help protect the muscle from injury under these conditions. Conversely, the greater resistance to stretch during the initial phase of stretching at lower temperatures may provide protection given the slower engagement of the titin-based stabilisation mechanism in the cold. It remains unclear whether the temperature sensitivity of muscle ‘*give*’ confers an adaptive functional benefit or simply reflects the different temperature sensitivities of the mechanisms underlying phases 1 and 2.

Intrinsic differences in muscle fibre type are likely to further shape the thermal sensitivity of this biphasic force response, given the baseline differences in cross-bridge kinetics and titin isoforms across phenotypically distinct muscles (9, 49). Bennett (9) reported that the Q10 of time to peak tension did not differ substantially among muscle types, whereas the temperature dependence of V_max_ was greater in faster muscles compared with slower ones. However, more recent work demonstrates that temperature sensitivity can be muscle-specific and vary regionally within an organism, indicating that thermal dependence is not necessarily uniform across all muscles (50).

### Implications for muscle modelling

Eccentric muscle contractions are an important component of muscle function during both cyclical locomotion and deceleration tasks. For example, during fish swimming, the slow muscle in the more posterior myotomes is recruited relatively earlier in the strain cycle, resulting in increased energy absorption (negative power) that is thought to stiffen the body and enhance power transfer to the tail (51, 52). Similarly, during landing in turkeys, the lateral gastrocnemius muscle fascicles are actively stretched and dissipate energy stored in the tendons during the initial phase of landing (53). Moreover, tissue thixotropy and muscle spindle firing are highly dependent on eccentric lengthening and rate of force development (28, 29). Therefore, accurately measuring and modelling muscle force during eccentric contractions is essential (54). However, current models of muscle of *in vivo* function remain relatively simplistic in their representation of the eccentric force-velocity relationship, often ignoring the biphasic force response that occurs during active stretch (23, 26, 55). This issue is highlighted by the varying temperature dependence of coefficients D and E, which are sensitive to the measurement location (Fig. 3, Table 3) and is likely to influence model estimates (56). This limitation partly reflects an incomplete understanding of the mechanisms that determine force during active lengthening, particularly their temperature dependence. This is important given that much of the available experimental data were obtained at non-physiological temperatures (Appendix Fig. A1) (14, 19, 21, 28, 29, 36, 37, 45, 46, 57–79). Our findings provide new insights into the eccentric force-velocity relationship and the temperature dependence of its underlying mechanisms, offering a framework for refining future models of muscle function under physiologically relevant conditions.

## Conclusion

Here we have shown, for the first time the temperature dependence of the muscle’s biphasic response to active stretching, providing new insight into the eccentric force-velocity. We observed a clear dichotomy in the temperature sensitivity of the rate of force development during phase-1 and phase-2, reflecting differences in the underlying mechanisms. The decrease in stiffness at higher temperatures during phase-1 suggests that cross-bridge detachment is accelerated at higher temperatures. However, the reduction in stiffness at cooler temperatures relative to normalised stretch velocity suggests that strain dependent detachment also contributes. In contrast, the increase in stiffness with temperature during phase-2 implies that higher temperatures promote stronger and faster titin-actin binding, increasing the proportion of titin molecules bound to the thin filament.

Furthermore, we have shown that muscle ‘*give*’ is not only a velocity dependent feature of active lengthening, but also a highly temperature sensitive phenomenon. The transition between phase-1 and phase-2 occurs at relatively higher forces at lower temperatures potentially underpinned by slower cross-bridge detachment, while the greater drop in force between phases suggests that there is slower engagement of the parallel elastic elements. Overall, the temperature dependence of the biphasic stretch response reflects an intrinsic tuning of muscle mechanics potentially to reduce the risk of damage under different thermal conditions. Together these findings provide new understanding of the muscle’s response to active stretch and its temperature dependence, providing a foundation for the development for more accurate musculoskeletal models of *in vivo* force.

## Supplementary Material

Supplementary Material

figure-7

figure-8

## Figures and Tables

**Figure:**
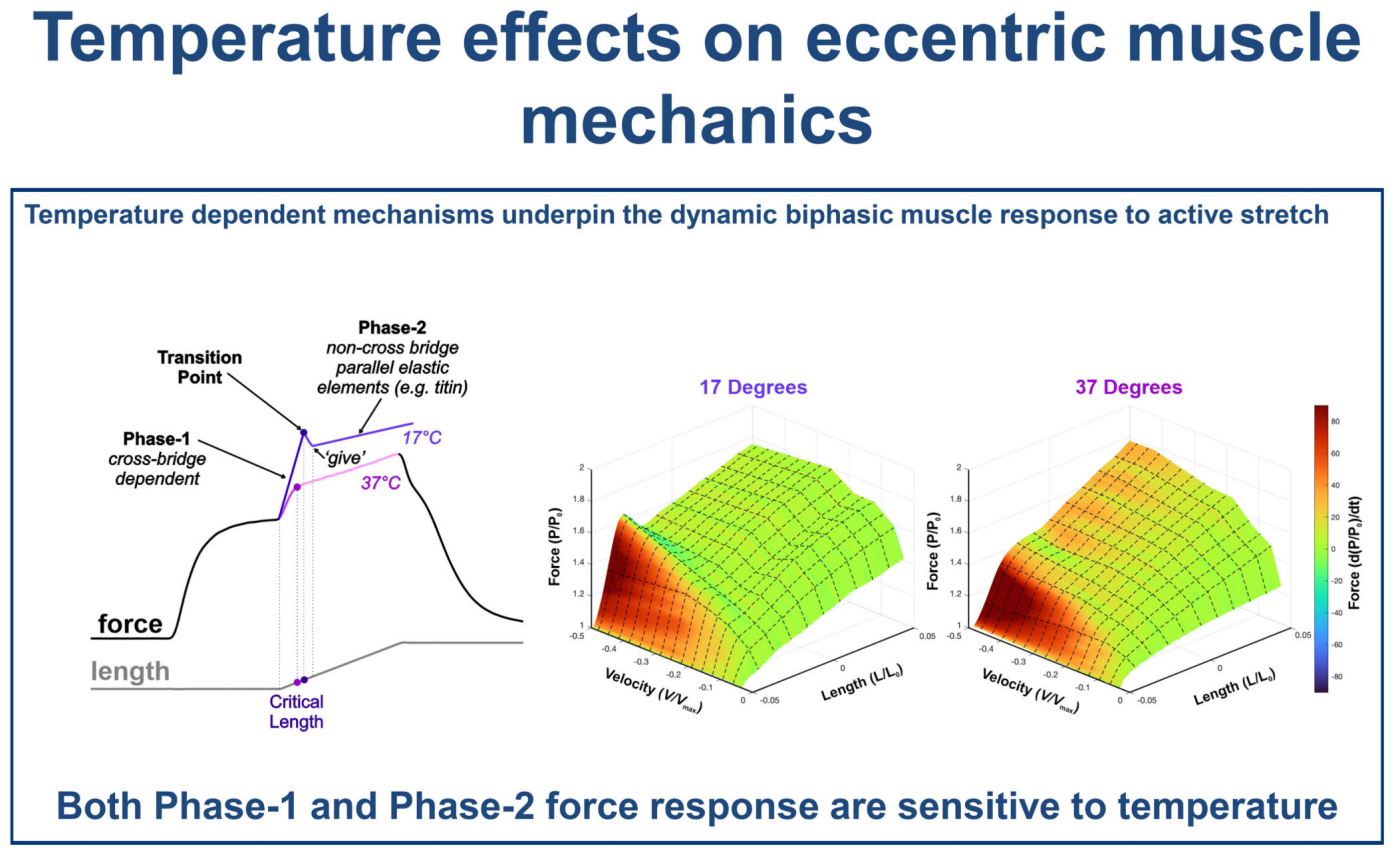


**Table 1:** Linear regression model statistics and pairwise statistics for the slope between the rates of force development and velocity of muscle lengthening across phase-1 and phase-2.

	Slopes [95% upper, lower confidence limits]			Model Fit	
	17°C	27°C	37°C	F	P value
Phase 1 (*d*P/P_0_/*dt*) *vs*. Absolute Velocity (*FL*.s^-1^)	-33.0 [-35.8, -30.2] ^§, #^	-16.8 [-18.2, -15.3] *	-15.7 [-16.5, -14.9] *	560.7	<0.001
Phase 1 (*d*P/P_0_/*dt*) *vs*. Normalised Velocity (*V/V*_max_)	-178 [-192, -163] ^#^	-185 [-201, -169] ^#^	-222 [-233, -210] *, ^§^	560.7	<0.001
Phase 2 (*d*P/P_0_/*dt*) *vs*. Absolute Velocity (*FL*.s^-1^)	-2.28 [-3.15, -1.40] ^#^	-3.26 [-3.70, -2.83] ^#^	-4.35 [-4.59, -4.11] *, ^§^	404.2	<0.001
Phase 2 (*d*P/P_0_/*dt) vs*. Normalised Velocity (*V/V*_max_)	-12.9 [-17.9, -7.94] ^§, #^	-36.0 [-40.8, -31.21] * ^#^	-61.5 [-64.9, -58.11] *, ^§^	404.2	<0.001

P value adjusted, Tukey method for comparing pairwise differences.

* P<0.05 *vs*. 17°C (n=5)

§ P<0.05 *vs*. 27°C (n=5)

# P<0.05 *vs*. 37°C (n=6).

**Table 2:** Isometric and Isotonic properties of the extensor digitorum longus in response to modified temperature.

	17°C	27°C	37°C	F	P value
***V*_max_**	5.66 ± 0.65^§ #^	11.05 ± 1.62* ^#^	14.14 ± 1.77* ^§^	51.727	<0.001
***W*_max_**	156.07 ± 9.36^§ #^	534.21 ± 159.51*	402.64 ± 108.15*	15.698	<0.001
***P/P_0_*at maximal power**	0.41 ± 0.02	0.42 ± 0.02^#^	0.39 ± 0.02^§^	4.546	0.023
***V/V***_max_ **at maximum power**	0.26 ± 0.02^#^	0.29 ± 0.03	0.33 ± 0.05*	6.883	0.005
**Power ratio**	0.11 ± 0.01^#^	0.12 ± 0.01	0.13 ± 0.01*	6.127	0.008
**A**	0.132 ± 0.037	0.167 ± 0.057	0.396 ± 0.295	3.836	0.038
**B**	0.548 ± 0.140	1.261 ± 0.330	5.560 ± 5.389	4.205	0.029
**C**	1.454 ± 0.393	3.290 ± 0.873	1.547 ± 3.019	1.551	0.235

V_max_; maximum shortening velocity, W_max_; maximum isotonic power. Coefficient A, B and C correspond to values for the Marsh and Bennett (1986) hyperbolic linear equation fit for the concentric portion of the force-velocity relationship presented in Equation 1. Mean value ± standard deviation. One-way ANOVAs were used to assess statistical significance, where significant main effects were detected, post hoc comparisons were conducted using the Bonferroni correction and the threshold for statistical significance set to P<0.05.

* P<0.05 *vs*. 17°C (n=5)

§ P<0.05 *vs*. 27°C (n=7)

# P<0.05 *vs*. 37°C (n=12).

**Table 3:** Eccentric force-velocity curve coefficients and their response to temperature in the extensor digitorum longus.

	17°C	27°C	37°C	F	P value
**Calculated crossing L_0_**					
**D**	-0.370 ± 0.048	-0.375 ± 0.140	-0.429 ± 0.038	0.814	0.464
**E**	0.162 ± 0.052	1.167 ± 1.071	0.869 ± 0.248	3.553	0.059
**Calculated at the transition point**					
**D**	-0.082 ± 0.167^§, #^	-0.283 ± 0.044*	-0.359 ± 0.034*	11.327	0.001
**E**	1.475 ± 0.605^§, #^	3.188 ± 1.305*	4.596 ± 0.744*	15.646	< 0.001

D and E correspond to value fit for the hyperbolic equation Kissane and Askew (22) to fit the eccentric portion of the force-velocity relationship, displayed in Equation 2. Mean value ± standard deviation. One-way ANOVAs were used to assess statistical significance, where significant main effects were detected, post hoc comparisons were conducted using the Bonferroni correction and the threshold for statistical significance set to P<0.05.

* P<0.05 *vs*. 17°C (n=5)

§ P<0.05 *vs*. 27°C (n=5)

# P<0.05 *vs*. 37°C (n=6).

## Data Availability

Source data for this study are openly available at: DOI:10.17638/datacat.liverpool.ac.uk/3052

## New & Noteworthy

Muscles sometimes work as brakes, generating force while they lengthen. We show this force rises in two phases both strongly affected by temperature, reflecting distinct underlying mechanisms. Phase-1 stiffness decreases at higher temperatures consistent with faster cross-bridge detachment. In contrast, phase-2 stiffness increases with temperature implying enhanced and more rapid titin-actin binding. These temperature-dependent changes reveal how muscles are tuned to resist active stretch, with potential implications for susceptibility to damage under varying thermal conditions.
