# Supplementary material for: How temperature tunes muscle mechanics during eccentric contractions
Source: Am J Physiol Cell Physiol. Author manuscript; Available in PMC 2026 Apr 4. (PMC7618963; doi:10.1152/ajpcell.00047.2026)
Supplement: Supplementary Material [file EMS213052-supplement-Supplementary_Material.docx]

## APPENDIX

**Appendix Figure A1. Overview of the temperature used in eccentric ramp experiments.** The temperatures used across 33 published manuscripts where eccentric muscle ramps have been quantified. Here the references have been categorised based on the muscle scale/preparation type, single sarcomere and single fibres (circle), *ex-vivo* fibre bundles (square) and *in situ* whole muscle (triangle). (14, 19, 21, 22, 36, 37, 45, 46, 57-81).

**Appendix Figure A2. Force-velocity and isometric metrics as a function of temperature.** Concentric force-velocity data are presented from this manuscript (circles) and Rummel’s et al. (2018) (triangles). Here we show the comparable temperature dependence of V_max_ (A) and the power ratio (B). Isometric twitch kinetics, mainly twitch rise time (C) and half-relaxation (D) also present with substantial temperature dependence. Further, here we show that the slope between lengthening velocity and the rate of force development of phase-1 and phase-2 have a strong temperature dependence (E). Finally, the D coefficient (plateau height) and E coefficient (curvature) of the eccentric force-velocity relationship also present with a temperature dependence. Values presented indicate the Q_10_ value between the selected temperature intervals.

Computational biomechanical models are often necessary to understand complex biological systems (e.g. masticatory system (23); locomotion: (24)), to explore optimal performance (25) or to predict *in vivo* function in biomechanics (26, 27) and motor control research (28, 29). These models must incorporate a representation of the eccentric force-velocity relationship. However, the experimental data underpinning such models are frequently derived from different species and recorded at non-physiological temperatures (Appendix Fig. A1), which may limit their physiological relevance. Here we have tabulated temperatures used to perform eccentric ramp experiments highlighting that much of the available experimental data are obtained at non-physiological temperatures (Appendix Fig. A1) (14, 19, 21, 28, 29, 36, 37, 45, 46, 57-79). The subsequent impact of temperature on isometric and force-velocity parameters is detailed in Appendix Fig. A2. Briefly, the temperature dependence of the concentric force-velocity relationship of our mouse EDL is similar to that reported by Rummel et al. (41) in the female, CD1 mouse EDL (Appendix Fig. A2 A-B). Isometric twitch kinetics (e.g. twitch rise time and half-relaxation time) present with the some of the greatest temperature sensitivities, with Q_10_ values as high as 4 (Appendix Fig. A3 C-D). We demonstrate that the relationship between the rate of relative force development and stretch velocity during phase-1 (the stiffness or the change in relative force per unit length) is temperature dependent. The slope being steeper at cooler temperatures, indicating greater stiffness, which is evidenced by Q_10_ values below 1: 0.51 for the 17°C to 27°C range and 0.93 for the 27°C to 37°C range (Appendix Fig. A2 E). Finally, the sensitivity of the coefficients derived from the eccentric force-velocity relationship present with a large temperature sensitivity (Appendix Fig A2F).
