# Supplementary material for: How temperature tunes muscle mechanics during eccentric contractions
Source: Am J Physiol Cell Physiol. Author manuscript; Available in PMC 2026 Apr 4. (PMC7618963; doi:10.1152/ajpcell.00047.2026)
Supplement: figure-7 [file EMS213052-supplement-figure_7.pdf]

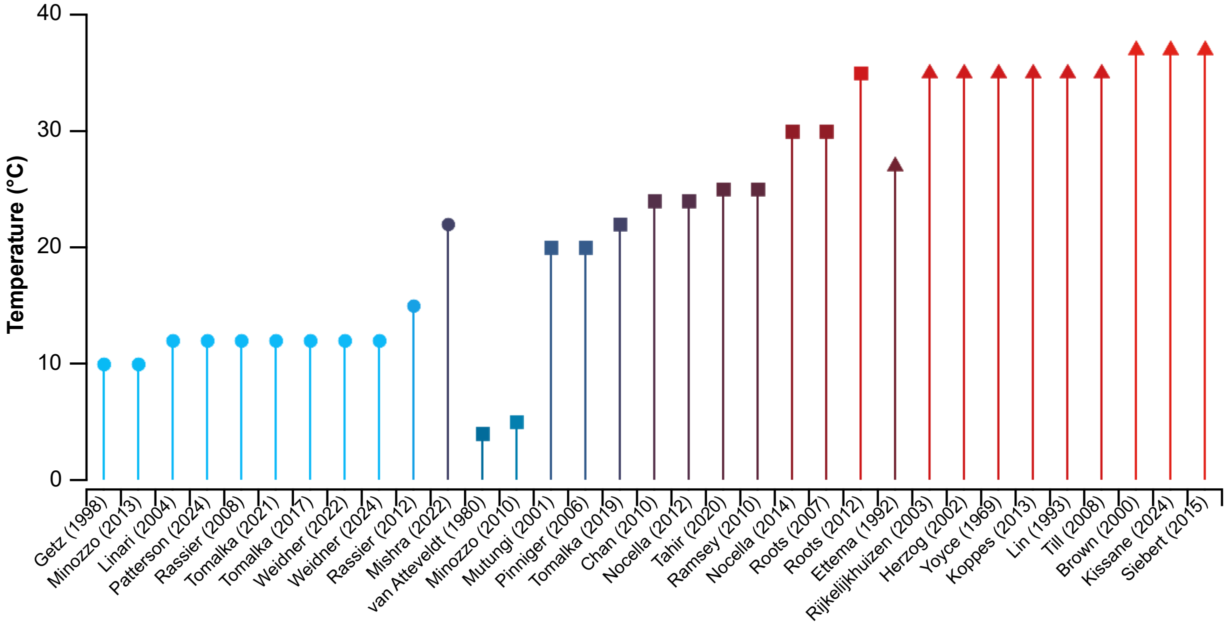

Appendix Figure 1. Overview of the temperature used in eccentric ramp experiments. The temperatures used across 33 published manuscripts where eccentric muscle ramps have been quantified. Here the references have been categorised based on the muscle scale/preparation type, single sarcomere and single fibres (circle), *ex-vivo* fibre bundles (square) and *in situ* whole muscle (triangle). (14, 19, 21, 22, 36, 37, 45, 46, 53-77).
