# Supplementary material for: How temperature tunes muscle mechanics during eccentric contractions
Source: Am J Physiol Cell Physiol. Author manuscript; Available in PMC 2026 Apr 4. (PMC7618963; doi:10.1152/ajpcell.00047.2026)
Supplement: figure-8 [file EMS213052-supplement-figure_8.pdf]

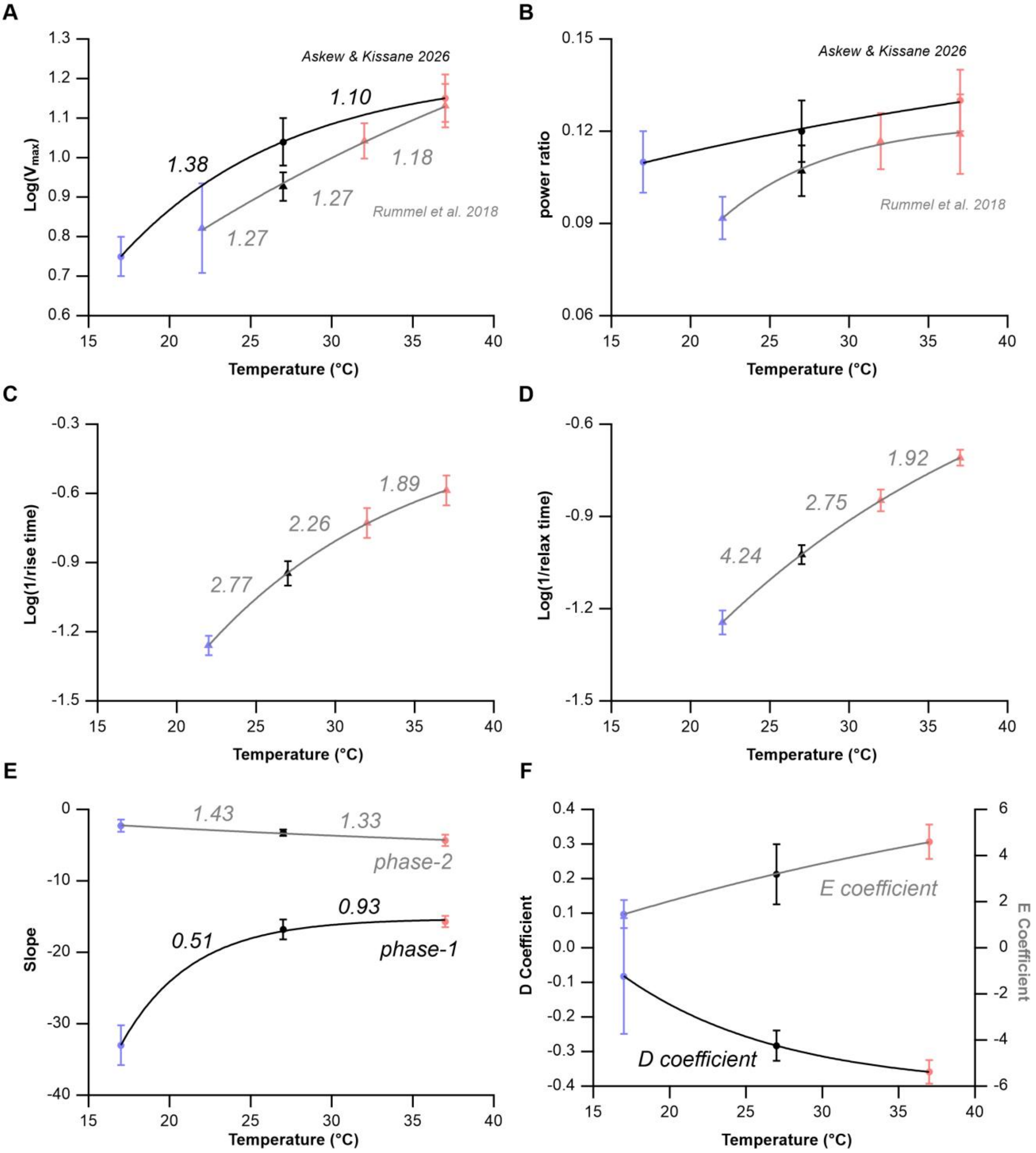

Appendix Figure 2. Force-velocity and isometric metrics as a function of temperature. Concentric force-velocity data are presented from this manuscript (circles) and Rummel's et al. (2018) (triangles). Here we show the comparable temperature dependence of V_{max} (A) and the power ratio (B). Isometric twitch kinetics, mainly twitch rise time (C) and half-relaxation (D) also present with substantial temperature dependence. Further, here we show that the slope between lengthening velocity and the rate of force development of phase-1 and phase-2 have a strong temperature dependence (E). Finally, the D coefficient (plateau height) and E coefficient (curvature) of the eccentric force-velocity relationship also present with a temperature dependence. Values presented indicate the Q_{10} value between the selected temperature intervals.
